# Pulmonary blood volume variation indexed to stroke volume and perfusion gradients: novel diagnostic tools in heart failure

**DOI:** 10.1186/1532-429X-18-S1-Q23

**Published:** 2016-01-27

**Authors:** Mariam Al-Mashat, Mikael Kanski, Jonas Jögi, Håkan Arheden

**Affiliations:** grid.4514.40000000109302361Department of Clinical Physiology, Skane University Hospital, Lund University, Lund, Sweden

## Background

Heart failure (HF) is a condition characterized by increased filling pressures in the left ventricle due to altered cardiopulmonary blood flow. However, there are no clinically available non-invasive quantitative methods to measure filling pressures in HF. The pulmonary blood volume variation (PBVV) indexed to stroke volume (SV) by cardiac magnetic resonance (CMR) could be a novel non-invasive measure of HF. However, the relationship between PBVV/SV and increased filling pressures has not been validated. We have previously shown that the effect of increased filling pressures and redistribution of pulmonary blood flow can objectively be quantified using perfusion gradients derived from ventilation/perfusion single-photon emission computed tomography (V/P SPECT). V/P SPECT perfusion gradients have a high positive predictive value to diagnose decompensated HF.

The aim of this study was therefore to investigate the relationship between the PBVV/SV and perfusion gradients in patients with heart failure.

## Methods

Fifteen patients with HF (NYHA class 2-4, 2 women, 48-77 years) were included. All patients underwent CMR at 1.5T and V/P SPECT. Four patients were under consideration for heart transplantation and 11 patients were candidates for cardiac resynchronization therapy. By measuring the blood flow in the pulmonary trunk and in one pulmonary vein, the PBVV was calculated, defined as the maximum cumulative difference in pulmonary blood volume over one heart beat. The SV by CMR was measured as the integral of the flow measurement over one heart beat in the pulmonary trunk. Dorso-ventral perfusion gradients (%-counts/cm) were automatically derived from V/P SPECT images using a user independent algorithm previously developed and validated in our department.

## Results

**Figure 1:**
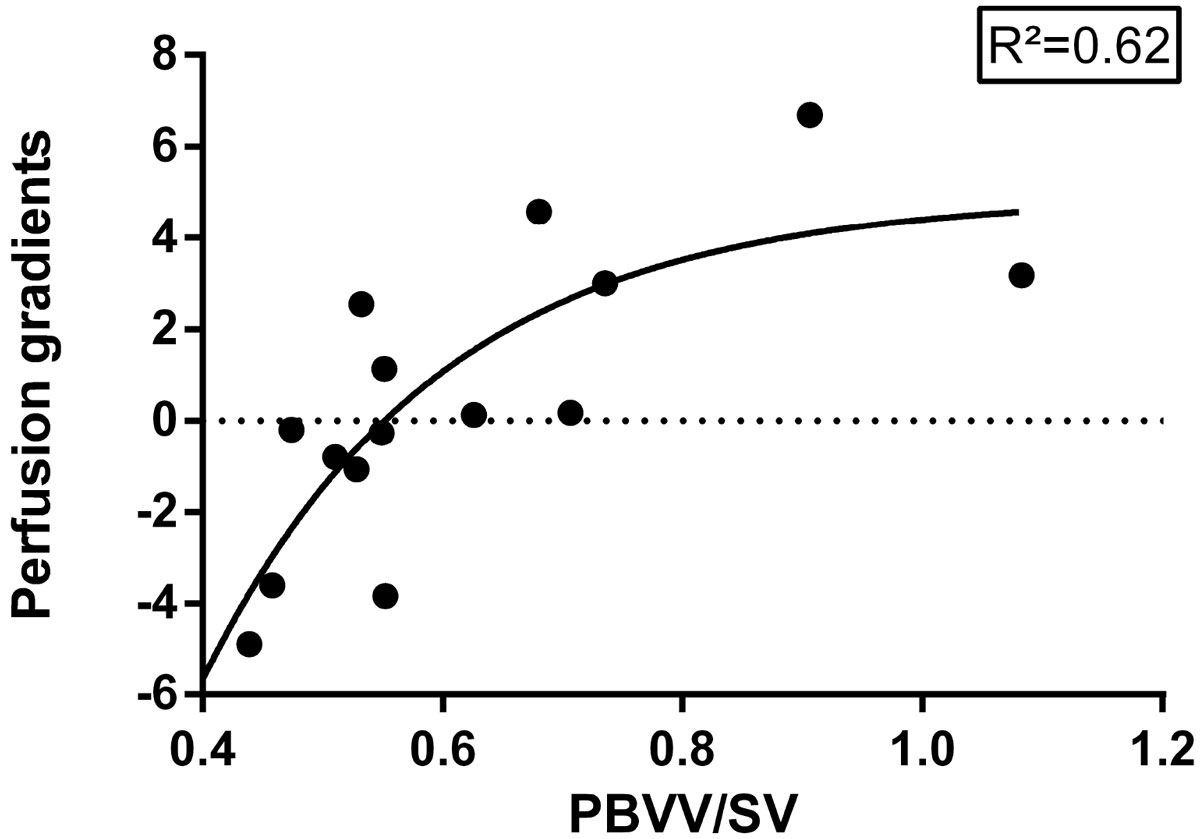
**Pulmonary blood volume variation indexed to stroke volume versus perfusion gradients in patients with moderate - severe heart failure**. The solid line represents the fitted exponential curve.

There was a positive relationship between PBVV/SV and perfusion gradients (Figure 1) suggesting that PBVV/SV could be valuable in the diagnosis of HF.

## Conclusions

PBVV/SV measured by CMR, as well as perfusion gradients by V/P SPECT have the potential to serve as non-invasive measures of HF. Further validation work with comparison to invasive pressure measurements are needed to assess the accuracy of the methods.

